# Serum and gingival crevicular fluid asprosin levels in obese and normal-weight individuals with and without periodontitis: a cross-sectional study

**DOI:** 10.1186/s12903-026-08249-y

**Published:** 2026-04-06

**Authors:** Buse Naz Buyukakcali Altay, Aysen Bodur, Emin Umit Bagriacik, Nihan Oruklu, Fusun Balos Toruner, Hulya Nur Sodan

**Affiliations:** 1 Department of Periodontology, Faculty of Dentistry, Gazi University, Ankara, Emek 06510 Türkiye; 2 Department of Immunology, Faculty of Medicine, Gazi University, Ankara, Türkiye; 3 Department of Endocrinology and Metabolism, Gazi University, Faculty of Medicine, Ankara, Türkiye

**Keywords:** Asprosin, Obesity, Periodontitis, Adipokines

## Abstract

**Background:**

Obesity and periodontitis are chronic inflammatory conditions linked by shared immunometabolic pathways. Although asprosin has been implicated in metabolic regulation and systemic inflammation, its role in the immunometabolic interaction between obesity and periodontal inflammation remains unclear. This study evaluated local and systemic levels of asprosin, tumor necrosis factor-α (TNF-α), and interleukin-6 (IL-6) in obese and normal-weight individuals with and without periodontitis.

**Materials and methods:**

This cross-sectional study included 60 individuals aged 18–65 years, categorized by body mass index (BMI) as normal weight (18.5–24.9 kg/m²) or obese (≥ 30 kg/m²). Participants were further classified as periodontally healthy or with periodontitis, forming four groups: normal-weight periodontally healthy (NH), obese periodontally healthy (OH), normal-weight periodontitis (NP), and obese periodontitis (OP) (*n* = 15 each). Plaque index, gingival index, probing pocket depth, clinical attachment level, and bleeding on probing were recorded. Gingival crevicular fluid (GCF) and serum samples were analyzed for asprosin, TNF-α, and IL-6 using an enzyme-linked immunosorbent assay. Intergroup comparisons were performed using a rank-based general linear model adjusted for age and sex.

**Results:**

Serum asprosin levels were lower in the NH group than in the NP (*p* = 0.025), OH (*p* < 0.001), and OP (*p* < 0.001) groups and were also lower in the NP group than in the OH (*p* = 0.006) and OP (*p* = 0.021) groups. GCF asprosin levels were lower in the NH group compared with the OH (*p* < 0.001) and OP (*p* = 0.017) groups, and were also lower in the NP group than in the OH (*p* = 0.042) and OP (*p* = 0.022) groups. IL-6 levels were higher in the OH group than in the NH group (*p* = 0.050), whereas serum TNF-α did not differ significantly among groups (*p* = 0.078). Although overall group differences were observed for GCF IL-6 (*p* = 0.014) and TNF-α (*p* = 0.008), overlap in interquartile ranges limited consistent pairwise comparisons.

**Conclusion:**

GCF and serum asprosin levels were higher in obese individuals than in normal-weight participants, while serum asprosin levels were higher in normal-weight individuals with periodontitis than in normal-weight periodontally healthy individuals. These findings are consistent with an association between asprosin levels, obesity, and periodontal status, indicating that obesity status should be considered when interpreting asprosin levels in periodontal conditions.

**Trial registration:**

The study was retrospectively registered on ClinicalTrials.gov on March 17, 2025 (Identifier: NCT06879951).

**Supplementary Information:**

The online version contains supplementary material available at 10.1186/s12903-026-08249-y.

## Introduction

 Periodontal diseases result from the host’s response to the pathogenicity of specific microorganisms in the microbial dental biofilm attached to the tooth surface [1]. Periodontitis is a complex, chronic inflammatory disease influenced by multiple host factors, including genetics, socioeconomic status, age, gender, and systemic diseases [2]. The World Health Organization (WHO) defines obesity as an abnormal or excessive accumulation of body fat that negatively impacts overall health, arising from multifactorial causes, including genetic, metabolic, endocrine, socioeconomic, and behavioral factors [3]. Additionally, a lack of physical activity, poor eating habits, and gender can also contribute to the development of obesity [4]. Periodontal diseases and obesity are interconnected inflammatory conditions that share common mechanisms of development [1]. The 2017 classification of periodontal diseases and conditions identified obesity as a systemic factor that influences periodontal health. Specifically, obesity is known to increase periodontal inflammation and upregulate the risk of developing periodontitis [1]. Numerous studies have explored this association; however, mechanistic understanding remains limited.

Adipose tissue secretes a variety of bioactive molecules termed adipocytokines, which exert effects at both local (autocrine/paracrine) and systemic (endocrine) levels [5]. It is hypothesized that the adipokines and cytokines released from adipose tissue contribute to the onset and progression of periodontal disease [6]. In obesity, adipokine secretion is dysregulated, with increased pro-inflammatory mediators contributing to chronic, low-grade systemic inflammation [7, 8]. This may exacerbate local inflammatory responses in periodontal tissues, thereby accelerating periodontal breakdown [7, 9]. Additionally, the increased systemic inflammatory burden observed in obese individuals contributes to modifications in the GCF composition, potentially augmenting the risk and severity of periodontitis [10, 11].

Adipocytokines, including leptin, resistin, adiponectin, spexin, and vascular endothelial growth factor (VEGF), influence inflammation. Inflammation-related cytokines, such as TNF-α, interleukins (IL-1β, IL-6, IL-8, and IL-10), and monocyte chemotactic protein-1 (MCP-1), are secreted by adipocytes [12]. Among these adipokines, asprosin, a novel hormone discovered in 2016, is gaining attention for its roles in metabolism and inflammation. This molecule plays a crucial role in stimulating the liver to release glucose into the bloodstream [13]. Research has shown that serum asprosin levels are increased in chronic inflammatory diseases such as type 2 diabetes mellitus (T2DM), polycystic ovary syndrome, and obesity [14–17]. To date, four studies have explored asprosin in the context of periodontal disease: one examined its levels in patients with ST-elevation myocardial infarction (STEMI) and periodontitis [18]; another investigated serum and salivary asprosin concentrations across BMI-stratified groups with periodontitis [19]; third compared asprosin levels in biological fluids between periodontally healthy and diseased individuals [20]. A more recent study assessed asprosin in plasma, saliva, and GCF among obese and non-obese patients with or without periodontitis [21]. Despite these emerging findings, the literature still provides limited and partly conflicting evidence, and studies specifically designed to clarify the association of asprosin in the interplay between obesity and periodontitis remain scarce.

TNF-α and IL-6 are implicated in inflammatory pathways relevant to both obesity and periodontal disease [22–25]. These cytokines were selected as reference inflammatory markers because they are widely used indicators of systemic and local inflammatory status and allow comparison of asprosin levels with established inflammatory mediators in both conditions. Since asprosin is a relatively novel biomarker, evaluating TNF-α and IL-6 alongside asprosin was intended to assess whether asprosin levels are associated with well-recognized inflammatory markers in obesity and periodontal disease.

This study aimed to evaluate local and systemic levels of asprosin, TNF-α, and IL-6 in obese and normal-weight individuals with and without periodontitis and to explore the association between asprosin, obesity, and periodontal inflammation. We hypothesized that obesity and periodontal inflammation are associated conditions and that asprosin levels differ according to obesity and periodontal status. In addition, asprosin levels would be associated with inflammatory biomarkers, including TNF-α and IL-6.

## Materials and methods

### Ethics approval and determination of sample size

This study was supported by the Gazi University Scientific Research Support Unit (Project Code: 8985). Ethical approval was obtained from the Gazi University Faculty of Dentistry Clinical Research Ethics Committee (Decision No: E-21071282), in accordance with the principles of the Declaration of Helsinki (1975), as revised in 2013. The study is registered on ClinicalTrials.gov (Identifier: NCT06879951).

The required sample size was determined using G*Power. Based on a previous study evaluating visfatin levels in GCF [26], a power analysis indicated that a minimum of 13 participants per group would be sufficient, yielding a total sample size of at least 52 individuals. This calculation was based on an effect size of f = 0.5, a significance level (α) of 0.05, and a desired statistical power of 81.914%. To account for potential data loss or unforeseen complications during sample collection, the final sample size was increased to 15 participants per group. At the time of study planning, no previous studies had reported comparable GCF asprosin data across similar periodontal and obesity-based groupings. Therefore, the sample size calculation was based on effect-size estimates derived from published differences in GCF visfatin levels using a comparable study design. Visfatin was selected as the reference adipokine due to its well-established role in obesity-related inflammation and periodontal disease. Asprosin was prespecified as the primary biomarker outcome, whereas analyses of IL-6 and TNF-α were considered secondary and exploratory.

### Study design

Study planning, patient recruitment, treatments, and sample collection were performed at the clinics of the Department of Periodontology, Faculty of Dentistry, Gazi University, Türkiye, between September 2023 and June 2024. Participants were recruited from patients attending the periodontology clinic for periodontal examination and treatment during the study period. Individuals who met the eligibility criteria and agreed to participate were consecutively enrolled in the study. To exclude individuals with diabetes, patients were referred to the endocrinology department for assessment of hemoglobin A1c (HbA1c) and fasting blood glucose (FBG), along with BMI measurements (kg/m²). BMI was calculated using a calibrated scale (Health o meter^®^ 502KL, USA). According to the WHO classification, BMI values between 18.50 kg/m² and 24.99 kg/m² are considered normal, and values ≥ 30 kg/m² are classified as obese [27]. Patients meeting the eligibility criteria were invited to participate in the study.

### Inclusion and exclusion criteria

Inclusion criteria: (1) Willingness to participate in the study, (2) Inclusion required both HbA1c < 5.7% and fasting blood glucose (FBG) < 100 mg/dL, in accordance with the American Diabetes Association criteria [28] (3) No periodontal treatment or periodontal surgery in the last 6 months, (4) Patients with periodontitis stage 2, 3, and 4 or periodontally healthy status, (5) Patients aged between 18 and 65, (6) Systemically healthy individuals, except for obesity, according to medical history. Exclusion criteria:1) Pregnancy or lactation for female patients, 2) Use of antibiotics in the last 6 months, 3) Systemic conditions known to influence periodontal inflammation or metabolic status (e.g., cardiovascular diseases, hypertension, chronic inflammatory diseases, autoimmune or immunological disorders), 4) Current smokers or individuals with a smoking history within the past 6 months.

Individuals who met the study criteria were divided into four groups based on their periodontal status: Normal weight and periodontally healthy (NH = 15), normal weight and periodontitis (NP = 15), obese and periodontally healthy (OH = 15), and obese and periodontitis (OP = 15).

### Study population

Comprehensive periodontal and radiographic examinations were performed for all participants. Periodontitis was diagnosed and classified according to the 2018 EFP/AAP classification [29]. Staging was determined based on the severity and extent of periodontal tissue destruction, using CAL, PPD, and radiographic bone loss assessed on periapical and/or panoramic radiographs. Stage II periodontitis was defined by interdental CAL of 3–4 mm, Stage III by interdental CAL ≥ 5 mm with probing depths ≥ 6 mm and/or vertical bone loss, and Stage IV by severe attachment loss with additional complexity factors such as extensive bone loss or tooth loss attributable to periodontitis. All periodontitis cases included in the study were classified as generalized, defined as involvement of more than 30% of teeth [29]. Periodontal health was defined as the absence of clinical attachment loss, probing pocket depth ≤ 3 mm, and bleeding on probing < 10% [30].

Full-mouth clinical parameters were recorded at four sites per tooth (mesial, buccal, distal, and lingual/palatal). For site-specific analyses, measurements were obtained from the sites at which GCF samples were collected. The recorded clinical parameters included probing pocket depth (PPD, mm), bleeding on probing (BOP) [31], plaque index (PI) [32], and clinical attachment level (CAL). All clinical measurements were performed using a periodontal probe (UNC-12, 1–12 mm; DuraLite^®^ ColorRings™, NordentColorRings™, Nordent). Information on oral hygiene habits was obtained using a structured questionnaire administered at the time of the clinical examination. Participants were asked about tooth brushing frequency, interdental cleaning practices, and dental visit frequency. Tooth brushing frequency was categorized as ≥ 2 times per day. Interdental cleaning was recorded as present or absent based on the self-reported use of dental floss or interdental brushes. Dental visit frequency was categorized as regular (at least once per year) or irregular.

All clinical measurements were performed by a single calibrated examiner (BNBA). Prior to the commencement of the study, intra-examiner reliability was assessed using a calibration sample of 10 individuals diagnosed with periodontitis who declined participation in the main study. Within 24 h, CAL and PPD were measured twice at four sites per tooth for each participant. Reproducibility was confirmed when the percentage of agreement within a 1 mm threshold between repeated measurements reached at least 90% for both CAL and PPD, in accordance with established calibration standards.

### Collection of GCF and serum samples

Venous blood samples were collected between 08:30 and 09:00 in the morning following an overnight fasting period of eight hours [33]. Venous blood samples (5 mL) were collected from each participant by standard antecubital venipuncture using sterile vacuum blood collection systems (BD Vacutainer^®^, Becton, Dickinson and Company, Franklin Lakes, NJ, USA). Blood was drawn into serum separator tubes (BD Vacutainer^®^ SST™ tubes, catalog no. 367986), without anticoagulant. The samples were centrifuged at 1,500 × g for 10 min to separate serum, which was then stored at − 80 °C until the day of analysis [34].

For GCF collection, a 30-second sampling protocol was used [35]. For each participant, four paper strips were collected, and the target sites were isolated with sterile cotton rolls. Supragingival plaque and debris were carefully removed using sterile curettes. The area was then gently dried with an air-water spray. GCF samples were obtained from sites with the deepest probing pocket depth in patients with periodontitis. In periodontally healthy individuals, samples were collected from the maxillary anterior teeth to minimize the risk of salivary contamination. Paper strips that were contaminated with blood or saliva were excluded from the study. The volume of GCF was measured using a Periotron device (Periotron 8000, Harco Electronics, Winnipeg, Canada). All GCF paper strips obtained from each participant were pooled into a single pre-labeled 2 mL polypropylene microcentrifuge tube (Eppendorf) and stored at − 80 °C until biochemical analysis.

### Biochemical analysis

The concentrations of asprosin, IL-6, and TNF-α in GCF and serum samples were determined using commercial ELISA kits. Thawed serum samples were used directly. To extract GCF samples, 200 µL of phosphate-buffered saline (PBS, pH 7.2) was added to each Eppendorf tube containing periopaper strips [36]. The GCF samples were vortexed for 3 min and then centrifuged at 8000 × g for 10 min using a microcentrifuge (Eppendorf AG, 5415D Centrifuge, Hamburg, Germany). All ELISA kits were prepared and performed according to the manufacturer’s instructions. The optical density (OD) was measured at 450 nm using a microplate reader (BioTek Int., Synergy HT, USA), and the concentrations of asprosin, IL-6, and TNF-α were calculated from the corresponding standard curves. The sensitivity of the kits was as follows: Abcam Human Asprosin ELISA Kit (Catalog no: ab275108) – 0.92 ng/mL; BT Lab TNF-α ELISA Kit (Catalog no: E0082Hu) – 1.52 ng/L; BT Lab IL-6 ELISA Kit (Catalog no: E0090Hu) – 1.03 ng/L. Concentrations reported by the ELISA kit in ng/L were converted to ng/mL.

GCF biomarker concentrations (ng/mL or ng/L) were converted to the total amount collected over 30 s (ng/30 s) using the equation described by Wassall and Preshaw [35].

### Statistical analysis

All statistical analyses were performed using IBM SPSS Statistics Standard Concurrent User Version 26 (IBM Corp., Armonk, NY, USA). Descriptive statistics were presented as frequencies (n) for categorical variables and as medians with interquartile ranges (IQRs) for continuous variables. The normality of numerical data was assessed using the Shapiro–Wilk test; because most continuous variables did not follow a normal distribution, descriptive statistics were reported as medians and IQRs. Spearman correlation analyses were performed to evaluate the relationships between BMI and biomarker levels, including serum and GCF asprosin, TNF-α, and IL-6, in the total study population. These correlation analyses were exploratory and were not adjusted for covariates. For unadjusted comparisons among the four study groups, the Kruskal–Wallis H test was applied for continuous variables, followed by Dunn–Bonferroni post hoc tests for multiple comparisons when appropriate. Associations between categorical variables were analyzed using the Chi-square test. To account for potential confounding effects of age and sex, adjusted analyses were performed using a rank-based general linear model, in which dependent variables were transformed into ranks. Separate models were constructed for each dependent variable (serum asprosin, GCF asprosin, serum TNF-α, GCF TNF-α, serum IL-6, and GCF IL-6). In each model, the study group was included as a fixed factor, and age and sex were included as covariates. All dependent variables were rank-transformed prior to analysis due to non-normal distribution. Group differences were evaluated while controlling for age and sex, and pairwise comparisons were conducted using estimated marginal means with Holm adjustment for multiple testing to control the type I error rate. A two-sided p-value < 0.05 was considered statistically significant.

## Results

A total of 60 individuals were included in the study. All participants were recruited from the same geographic region and were of Turkish ethnicity. Demographic characteristics of the participants, BMI, full-mouth and regional periodontal parameters, and GCF volumes are presented in Table 1. All periodontitis cases were classified as generalized periodontitis. In the NP group (*n* = 15), Stage II, III, and IV cases numbered 2, 9, and 4, respectively, whereas in the OP group (*n* = 15), the corresponding numbers were 5, 9, and 1.

**Table 1:** Description of the study population together with the site-level and full-mouth clinical parameters. GCF values (µl) are presented as median values with interquartile ranges (IQR)

Variables (Median, IQR)				NP (*n* = 15)	OH (*n* = 15)	OP (*n* = 15)	*p*
Age (Median, IQR)				46 (15)^a^	24 (9)^b^	54 (13)^a, c^	< 0.001^*^
Sex (n)		Female	10	9	8	9	0.556
		Male	5	6	7	6	
BMI (kg/ m²) (Median, IQR)				24.4 (2.30)	33.2 (2.5) ^a, b^	36.3 (4.10) ^a, b^	< 0.001^*^
Full-Mouth Clinical Measurements	PI (%)		9.8 (1)	40.78 (75)^a^	9.53 (5)^b^	70 (43)^a, c^	< 0.001^*^
	GI		0.21 (0.85)	0.78 (0.64) ^a^	0.24 (0)^b^	1.32 (1)^a, c^	< 0.001^*^
	PPD (mm)		1.41 (0)	2.90 (1)^a^	1.74 (0)^b^	2.85 (0.57) ^a, c^	< 0.001^*^
	CAL (mm)		0	2.35 (1)	0	1.98 (1)	< 0.001^*^
	BOP (%)		8.9 (3.93)	48.9 (65.4) ^a^	9.75 (2.46) ^b^	59.8 (28.3) ^a, c^	< 0.001^*^
Site-Level Clinical Measurements	VPI (%)		0	100 ^a^	0^b^	100 ^a, c^	< 0.001^*^
	GI		0	2^a^	0^b^	2 ^a, c^	< 0.001^*^
	PPD (mm)		2 (1)	7 (2)^a^	1.5 (1)^b^	7 (1) ^a, c^	< 0.001^*^
	CAL (mm)		0	7 (2) ^a^	0 ^b^	6 (2) ^a, c^	< 0.001 ^*^
	BOP (%)		0	100 ^a^	0^b^	100 ^a, c^	< 0.001 ^*^
GCF Volume (µl)				1.5 (0.5)^a^	0.63 (0.6)^b^	2.1 (0.67) ^a, c^	< 0.001 ^*^

Values are presented as median (interquartile range) unless otherwise stated. Group comparisons for continuous variables were performed using the Kruskal–Wallis test. When a significant overall difference was detected, pairwise comparisons were conducted using Dunn’s test with Bonferroni adjustment. Categorical variables were compared using the chi-square test. Different superscript letters indicate statistically significant differences between groups (*p* < 0.05)

n: sample size

*Abbreviations*: *BMI *Body Mass Index, *PI* Plaque Index, *GI* Gingival Index, *PPD* Probing Pocket Depth, *CAL* Clinical Attachment Level, *BOP* Bleeding on Probing

ᵃ = significantly different compared to healthy group, ᵇ = significantly different compared to normal weight periodontitis group, ᶜ = significantly different compared to obese healthy group

The participants’ oral hygiene habits are presented in Table 2. No statistically significant differences were observed among the study groups in tooth brushing frequency, interdental cleaning practices, or dental visit frequency (*p* > 0.05).

**Table 2:** Self-reported oral hygiene habits across the study groups

Variable	NH	NP	OH	OP	*p*-value
Tooth brushing ≥ 2/day n (%)	12 (80%)	13 (87%)	11 (73%)	10 (67%)	0.54
Interdental cleaning n (%)	6 (40%)	7 (47%)	5 (33%)	4 (27%)	0.61
Regular dental visits n (%)	5 (33%)	6 (40%)	4 (27%)	3 (20%)	0.48

Values are presented as number (n) and percentage (%). Categorical variables were compared using the chi-square test

*Abbreviations*: *NH *normal-weight periodontally healthy, *NP* normal-weight periodontitis, *OH* obese periodontally healthy, *OP* obese periodontitis. Statistical significance was defined as *p* < 0.05

Correlation analysis results are presented in Table 3. BMI showed strong positive correlations with both serum and GCF asprosin levels (*r* = 0.703 and *r* = 0.711, respectively; *p* < 0.001 for both). BMI was also positively correlated with GCF IL-6 levels (*r* = 0.286, *p* = 0.027). Serum asprosin levels showed significant positive correlations with serum IL-6 (*r* = 0.364, *p* = 0.004) and GCF asprosin levels (*r* = 0.607, *p* < 0.001). In addition, GCF asprosin levels were positively correlated with serum IL-6 (*r* = 0.316, *p* = 0.014) and GCF TNF-α levels (*r* = 0.262, *p* = 0.043). No significant correlations were observed between serum TNF-α levels and other biomarkers (*p* > 0.05).

**Table 3:** Spearman correlation matrix for BMI and serum and GCF levels of asprosin, TNF-α, and IL-6 in the overall study population (*n* = 60)

Variables	BMI	Serum Asprosin	Serum TNF-α	Serum IL-6	GCF Asprosin	GCF TNF-α	GCF IL-6
BMI	–	0.703 **(< 0.001)**	0.229 (0.079)	0.240 (0.064)	0.711 (< 0.001)	0.097 (0.459)	0.286 (0.027)
Serum Asprosin	0.703 (< 0.001)	–	0.132 (0.314)	0.364 (0.004)	0.607 (< 0.001)	0.088 (0.504)	0.221 (0.090)
Serum TNF-α	0.229 (0.079)	0.132 (0.314)	–	0.187 (0.153)	0.058 (0.660)	0.089 (0.500)	0.060 (0.650)
Serum IL-6	0.240 (0.064)	0.364 (0.004)	0.187 (0.153)	–	0.316 (0.014)	0.028 (0.830)	0.114 (0.380)
GCF Asprosin	0.711 (< 0.001)	0.607 (< 0.001)	0.058 (0.660)	0.316 (0.014)	–	0.262 (0.043)	0.249 (0.055)
GCF TNF-α	0.097 (0.459)	0.088 (0.504)	0.089 (0.500)	0.028 (0.830)	0.262 (0.043)	–	0.128 (0.330)
GCF IL-6	0.286 (0.027)	0.221 (0.090)	0.060 (0.650)	0.114 (0.380)	0.249 (0.055)	0.128 (0.330)	–

Spearman correlation coefficients (r) and corresponding p-values among BMI and serum and GCF levels of asprosin, TNF-α, and IL-6 in the total study population (*n* = 60). Values are presented as correlation coefficients (r) with p-values in parentheses

*Abbreviations*: *BMI *body mass index, *GCF* gingival crevicular fluid, *TNF-α* tumor necrosis factor-alpha, *IL-6* interleukin-6

Statistical significance was defined as *p* < 0.05

**Fig. 1:**
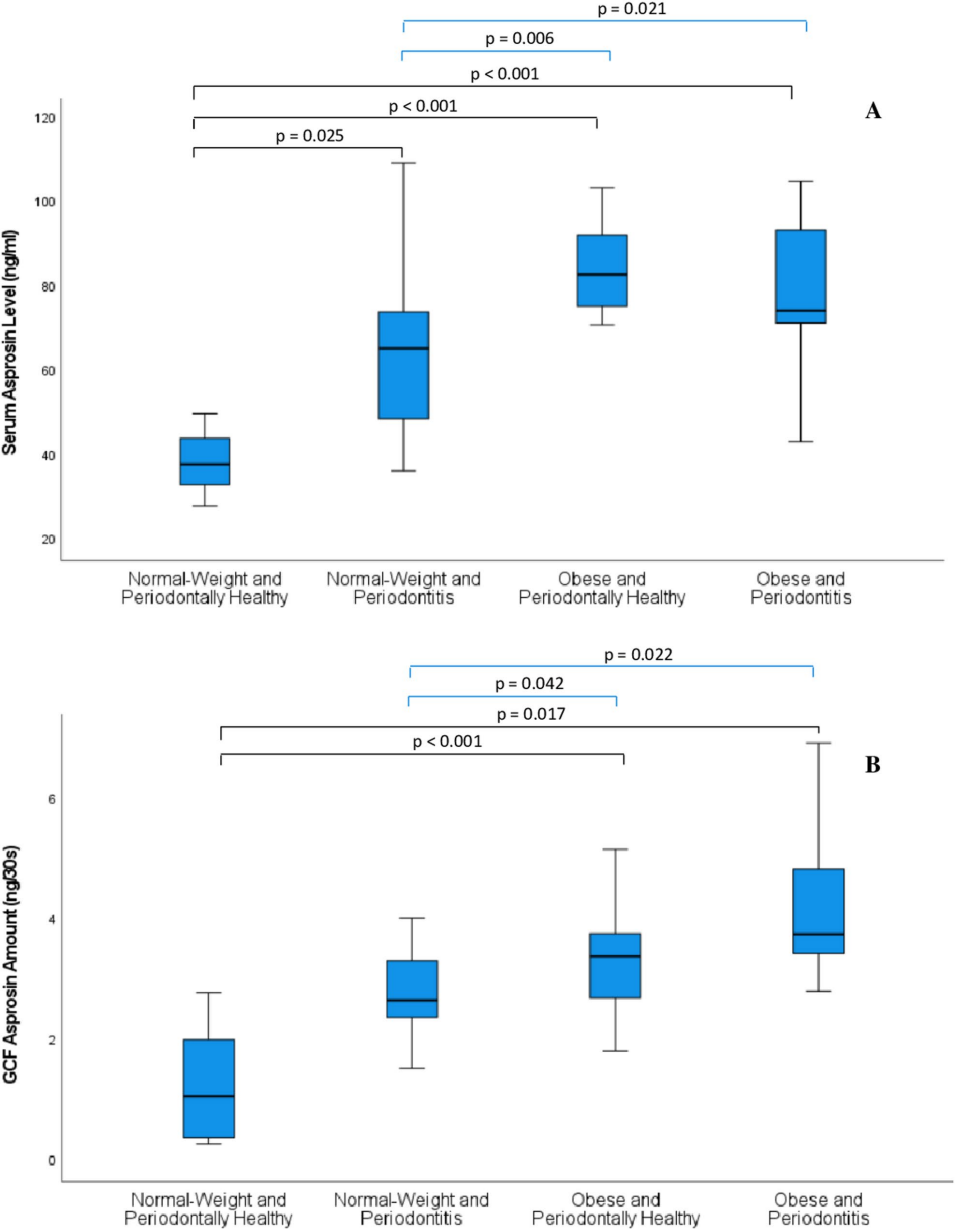
Serum and gingival crevicular fluid (GCF) asprosin levels across the study groups. **A** Serum asprosin levels (ng/mL). The NH group exhibited significantly lower median values compared with the NP (adjusted p = 0.025), OH (adjusted p < 0.001), and OP (adjusted p < 0.001) groups. In addition, significant differences were observed between the NP and OH groups (adjusted p = 0.006) and between the NP and OP groups (adjusted p = 0.021). **B **GCF asprosin amount (ng/30 s). GCF asprosin levels also differed significantly across the groups after covariate adjustment (p < 0.001). The NH group showed significantly lower median values compared with the OH (adjusted p < 0.001) and OP (adjusted p = 0.017) groups. Furthermore, significant differences were observed between the NP and OH groups (adjusted p = 0.042) and between the NP and OP groups (adjusted p = 0.022). Data are presented as median and interquartile range (boxplots). Group comparisons were performed using rank-based general linear models adjusted for age and sex, with Holm correction for multiple comparisons. NH, normal-weight periodontally healthy; NP, normal-weight periodontitis; OH, obese periodontally healthy; OP, obese periodontitis

After adjustment for age and sex, serum asprosin levels differed significantly among the groups (*p* < 0.001). The NH group exhibited significantly lower median values compared with the NP (adjusted *p* = 0.025), OH (adjusted *p* < 0.001), and OP (adjusted *p* < 0.001) groups. In addition, significant differences were observed between the NP and OH groups (adjusted *p* = 0.006) and between the NP and OP groups (adjusted *p* = 0.021) (Fig. 1A). 

GCF asprosin levels also differed significantly across the groups after covariate adjustment (*p* < 0.001). The NH group showed significantly lower median values compared with the OH (adjusted *p* < 0.001) and OP (adjusted *p* = 0.017) groups. Furthermore, significant differences were observed between the NP and OH groups (adjusted *p* = 0.042) and between the NP and OP groups (adjusted *p* = 0.022) (Fig. 1B).

**Fig. 2:**
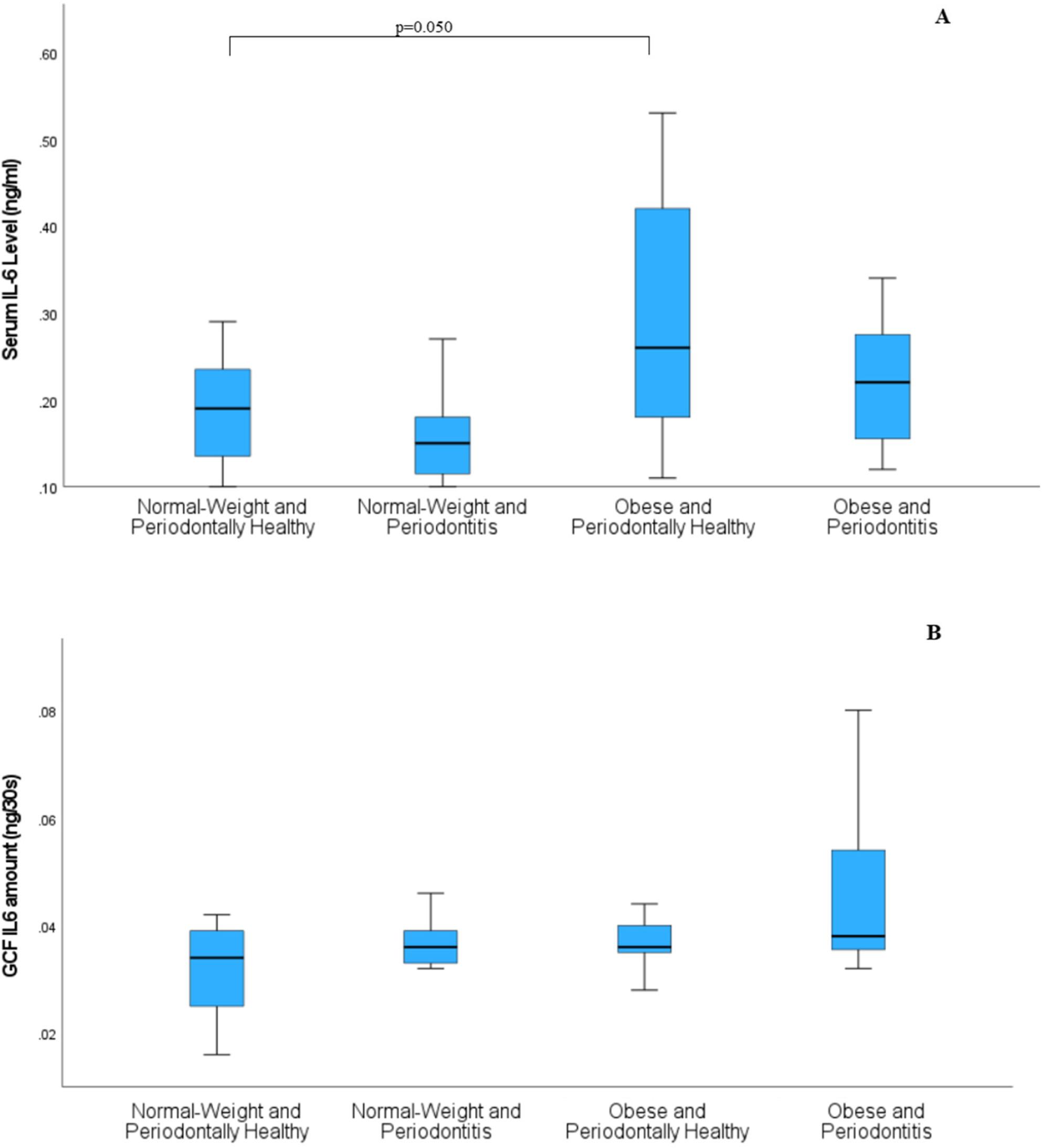
Serum and gingival crevicular fluid (GCF) interleukin-6 (IL-6) levels across study groups. **A **Serum IL-6 levels (ng/mL). A significant overall group difference was observed after adjustment for age and sex (p = 0.021), with the obese periodontally healthy (OH) group showing higher median values than the normal-weight periodontally healthy (NH) group, reaching marginal statistical significance after adjustment (adjusted p = 0.050). **B **GCF IL-6 amount (ng/30 s). Although an overall group difference was observed after covariate adjustment (p=0.014), substantial overlap in interquartile ranges limited the identification of consistent pairwise differences. Data are presented as medians and interquartile ranges (boxplots). Group comparisons were performed using rank-based general linear models adjusted for age and sex, with Holm correction for multiple comparisons. NH, normal-weight periodontally healthy; NP, normal-weight periodontitis; OH, obese periodontally healthy; OP, obese periodontitis

**Fig. 3:**
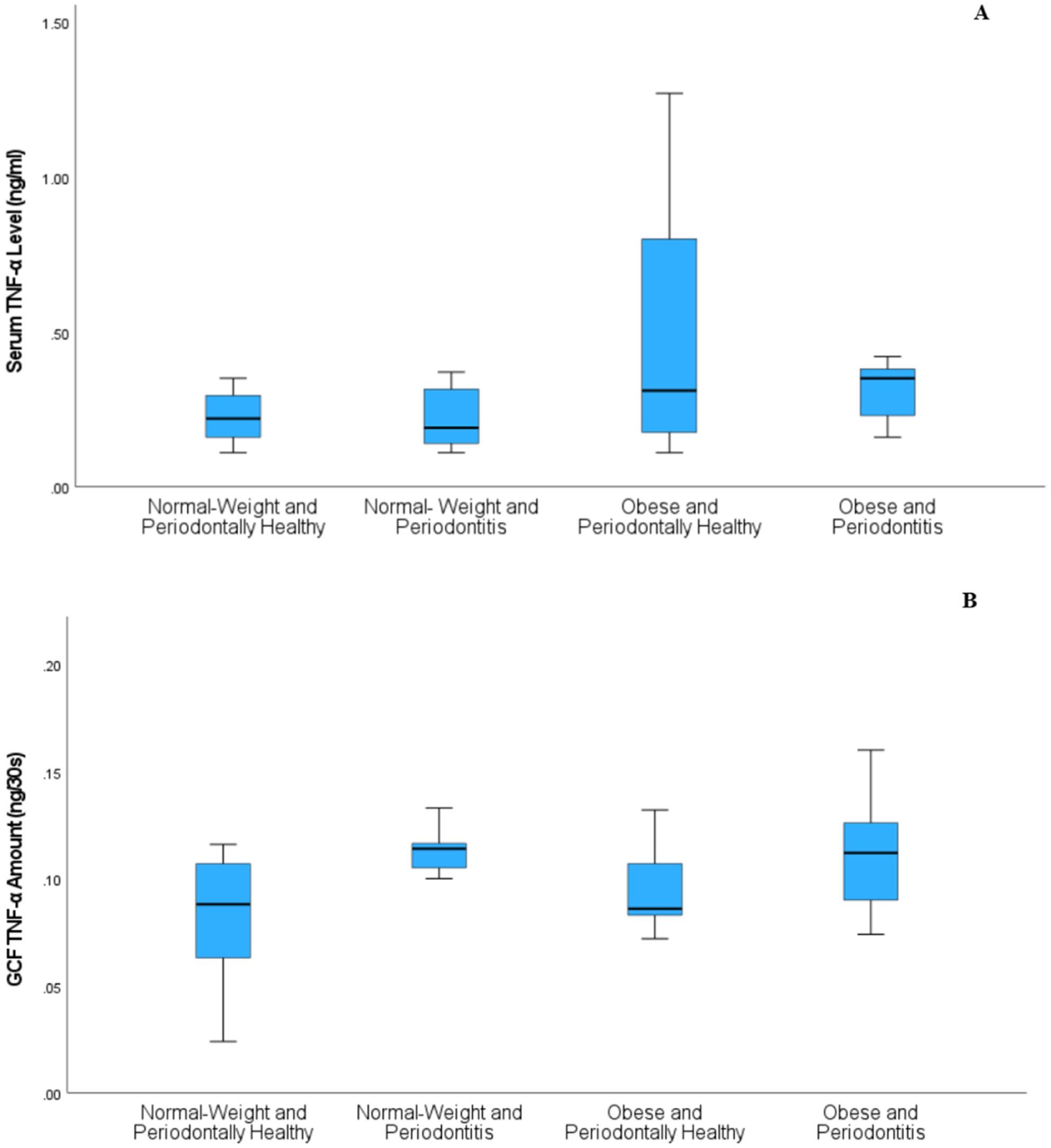
Serum and gingival crevicular fluid (GCF) tumor necrosis factor-α (TNF-α) levels across study groups. **A **Serum TNF-α levels (ng/mL). No significant differences were observed among the groups after adjustment for age and sex (p = 0.078). **B **GCF TNF-α amount (ng/30 s). Although an overall group difference was detected after covariate adjustment (p = 0.008), substantial overlap in interquartile ranges limited the identification of consistent pairwise differences. Data are presented as medians and interquartile ranges (boxplots). Group comparisons were performed using rank-based general linear models adjusted for age and sex, with Holm correction for multiple comparisons. NH, normal-weight periodontally healthy; NP, normal-weight periodontitis; OH, obese periodontally healthy; OP, obese periodontitis

Serum IL-6 levels demonstrated a significant overall group difference after adjustment for age and sex (*p* = 0.021), with the OH group showing higher median values than the NH group, with a borderline adjusted p-value (*p* = 0.050) (Fig. 2A). In contrast, serum TNF-α levels did not differ significantly among the groups (*p* = 0.078) (Fig. 3A).

Although overall group differences were observed for GCF TNF-α (*p* = 0.008) (Fig. 3B) and GCF IL-6 (*p* = 0.014) (Fig. 2B), substantial overlap in interquartile ranges was noted across the NH, NP, OH, and OP groups. This overlap indicates considerable inter-individual variability, allowing detection of global group effects while limiting the identification of consistent pairwise differences. Descriptive statistics for all biomarkers are presented in (Table 4). 

**Table 4:** Serum and GCF biomarker levels across study groups

Variables (Median, IQR)		NH (*n* = 15)	NP (*n* = 15)	OH (*n* = 15)	OP (*n* = 15)	*p*
GCF Total Biomarker Levels (ng/ 30 s)	Asprosin	1.03 (1.66) [1.97–2.76]	2.63 (1.11) [2.21–5.37]	3.36 (1.39) ^a, b^ [1.79–5.14]	3.73 (1.58) ^a, b^ [2.78–6.91]	< 0.001^*^
	TNF-α	0.09 (0.05) [0.02–0.12]	0.11 (0.01) [0.08–0.14]	0.09 (0.03) [0.07–0.14]	0.1 (0.04) [0.07–0.18]	0.008^*^
	IL-6	0.03 (0.02) [0.02–0.04]	0.04 (0.01) [0.03–0.05]	0.04 (0.01) [0.03–0.04]	0.04 (0.03) [0.03–0.09]	0.014^*^
Serum Biomarker Levels (ng/mL)	Asprosin	37.4 (13.11) [27.49–49.37]	64.8 (31.27) ^a^ [35.80-108.90]	82.4 (18.6) ^a, b^ [70.48–103]	73.85 (28.53) ^a, b^ [42.74–104.5]	< 0.001^*^
	TNF-α	0.22 (0.15) [0.09–0.55]	0.19 (0.20) [0.11- 0.36]	0.31 (0.84) [0.11- 0.89]	0.35 (0.17) [0.16–0.42]	0.078
	IL-6	0.16 (0.11) [0.08–0.29]	0.15 (0.09) [0.10–0.30]	0.26 (0.27) ^a^ [0.11–0.53]	0.22 (0.13) [0.12–0.34]	0.021^*^

Values are presented as median (interquartile range), with minimum and maximum values shown in square brackets [min–max]. Group comparisons were performed using rank-based general linear models adjusted for age and sex. The p-values represent adjusted global group comparisons, with pairwise comparisons corrected using the Holm method. Superscript letters indicate statistically significant differences between groups based on adjusted pairwise comparisons (*p* < 0.05)

*Abbreviations*: *NH *normal-weight periodontally healthy, *NP *normal-weight periodontitis, *OH *obese periodontally healthy, *OP *obese periodontitis

n: sample size

*p < 0.05 = statistically significant

ᵃ = significantly different compared to NH group, ᵇ = significantly different compared to NP group

In the adjusted models, age was identified as a statistically significant covariate for GCF asprosin levels, whereas age and sex did not significantly affect serum asprosin levels or the other investigated biomarkers. Unadjusted analyses yielded largely comparable patterns, and minimum and maximum values for all variables are provided in Supplementary Table S1.

## Discussion

To our knowledge, this is among the first clinical study to examine both serum and GCF asprosin in relation to periodontitis and obesity simultaneously. The parallel evaluation of systemic and local compartments enabled a more integrated descriptive assessment of the potential association between asprosin and the interplay between obesity and periodontal inflammation.

The pattern of higher GCF asprosin levels in obese individuals compared with normal-weight participants may reflect an association between obesity status and local asprosin availability within the periodontal environment. Although asprosin is considered primarily of systemic origin, elevated GCF levels raise the possibility that local periodontal tissues or infiltrating immune cells may contribute indirectly to its presence, for example, through diffusion, local retention, or inflammatory microenvironment–related mechanisms [20]. Previous reports indicate that asprosin secretion may also arise from non-adipose tissues, including pancreatic and hepatic cells, under inflammatory conditions [37]. However, direct evidence of asprosin expression in gingival or periodontal cells remains lacking, representing a critical mechanistic gap to be addressed in future molecular or histological studies.

We found that serum asprosin levels were higher in normal-weight individuals with periodontitis than in normal-weight periodontally healthy participants, consistent with an association between systemic periodontal disease and inflammatory burden [38]. The elevation of serum asprosin observed in periodontitis may indicate mechanisms that disrupt metabolic homeostasis, although metabolic stress cannot be inferred from the present data.

Elevated asprosin levels observed in obese individuals, even in the absence of clinical periodontitis, may reflect obesity-related low-grade inflammation. These findings indicate that higher adiposity is associated with elevated systemic and local asprosin levels, even in the absence of clinical periodontitis. Although both obesity and periodontitis were associated with elevated serum asprosin levels, no statistically significant difference was observed in serum asprosin levels when the two conditions coexisted compared with OH. Both conditions are characterized by chronic low-grade inflammation and adipokine dysregulation [39, 40], which may converge on similar regulatory mechanisms of asprosin.

Moreover, the OH and OP groups exhibited significantly higher serum asprosin levels than the NP group. This pattern suggests that serum asprosin levels are more consistently aligned with obesity status than with periodontal status in this cohort. In contrast to our findings, a study [21] reported decreased serum and GCF asprosin levels in obese, periodontally healthy individuals, suggesting a compensatory metabolic phase. These discrepancies may be due to several factors, including population differences (e.g., ethnicity, age distribution, and obesity phenotype), variation in disease definitions, and diagnostic thresholds.

Asprosin, secreted by white adipose tissue, modulates metabolic processes including glucose homeostasis [41]. Elevated serum asprosin levels in obesity have been linked to hepatic glucose production and insulin resistance, as well as to broader metabolic dysregulation [42]. By restricting overt dysglycemia (HbA1c < 5.7%, FBG < 100 mg/dL), we minimized significant metabolic confounding. In the present study, systemic inflammatory markers showed limited and heterogeneous differences across the study groups. Serum TNF-α levels did not differ significantly between groups, whereas serum IL-6 levels demonstrated a modest difference only between normal-weight healthy and obese healthy participants. Although obesity is commonly associated with low-grade systemic inflammation, such changes may not invariably appear as measurable elevations in circulating cytokines [43, 44]. These findings likely reflect inter-individual variability, differences in metabolic health among obese individuals, and the possibility that inflammatory activity is compartmentalized within tissues rather than being uniformly reflected in serum. In addition, metabolically healthy obesity, characterized by a relative predominance of M2 macrophages and regulatory T cells, may produce a balanced cytokine milieu [45]. Collectively, these considerations suggest that systemic cytokine levels may not consistently reflect inflammatory activity within adipose or periodontal tissues, underscoring the value of assessing both local and systemic compartments when investigating obesity-related inflammation.

At the local level, overall group differences were detected for both GCF TNF-α and IL-6; however, substantial overlap in interquartile ranges limited the identification of distinct pairwise differences between specific groups. This pattern reflects considerable inter-individual variability and suggests that local inflammatory activity may be shaped by multiple contextual factors beyond categorical obesity or periodontal status alone. The differing patterns between serum and GCF TNF-α and IL-6 levels may reflect the partially independent regulation of local and systemic inflammatory responses. GCF primarily represents site-specific inflammatory activity within periodontal tissues, whereas circulating cytokine levels are influenced by multiple systemic factors, including metabolic status and individual variability [46]. Consequently, inflammatory changes in periodontal tissues may not always be directly reflected in systemic cytokine levels.

Correlation analysis revealed a strong association between BMI and asprosin levels, supporting the association between obesity and increased asprosin expression. Correlations between asprosin and IL-6 may indicate inflammatory processes related to obesity and periodontal disease, while the correspondence between serum and GCF asprosin levels supports consistency between systemic and local measurements. In contrast, TNF-α showed weaker correlations with other biomarkers, suggesting a more complex inflammatory pattern. Correlation analyses were exploratory and were not adjusted for covariates; therefore, these findings should be interpreted cautiously.

TNF-α and IL-6 were included as reference inflammatory biomarkers to facilitate the interpretation of asprosin levels relative to established indicators of inflammation. The associations observed between asprosin and IL-6 support the relevance of evaluating novel biomarkers alongside conventional inflammatory mediators, although TNF-α showed more limited relationships. From a clinical perspective, the present findings suggest that obesity-related metabolic status could influence inflammatory biomarker levels in periodontal tissues even in the absence of clinically evident periodontitis. Therefore, obesity should be considered when interpreting biomarker-based assessments of periodontal inflammation.

A previous study demonstrated that both salivary and serum asprosin levels were markedly elevated in individuals with periodontitis [19]. In our study, we chose to analyze GCF instead of saliva, as GCF contains not only cells related to the local site and disease but also inflammatory products, offering more specific information about disease activity than the more generalized nature of saliva [47]. Also, in interpreting our findings, we considered the total amounts of the investigated biomolecules in GCF. The sample volume directly influences the concentrations of biomarkers in GCF (ng/µL).

A principal strength of this study is the parallel assessment of serum and GCF asprosin across four clinically distinct groups, allowing a more nuanced comparison of obesity- and periodontitis-related patterns. One study has compared GCF and serum asprosin levels in individuals with periodontally healthy, gingivitis, and periodontitis. Their results showed significant differences between the periodontitis and gingivitis groups compared to the periodontally healthy group [20].

Several limitations of this study should be acknowledged. First, a marked age imbalance was observed between the study groups. Although all analyses were adjusted for age and sex, residual confounding cannot be entirely excluded. Notably, the main findings regarding asprosin remained largely consistent after covariate adjustment, suggesting that the observed associations were not solely attributable to demographic differences. In contrast, group differences observed for TNF-α and IL-6 were attenuated after adjustment, indicating greater inter-individual variability and reinforcing the descriptive and hypothesis-generating nature of these findings.

At the time of study design, no prior data were available on asprosin levels in gingival crevicular fluid. Therefore, the sample size calculation was based on visfatin, a biologically related adipokine that has previously been investigated in periodontal inflammation. Although this approach was considered methodologically reasonable given the absence of prior asprosin data, it may have limited the statistical power for secondary biomarkers, which were therefore interpreted as exploratory outcomes.

Several potential confounding factors, including dietary habits, physical activity levels, socioeconomic status, hormonal status, and medication use, were not assessed. BMI was used as the primary indicator of obesity; however, it may not fully capture metabolic risk or body composition. Although BMI remains a widely used and accepted measure [48], its limitations underscore the importance of complementary metabolic assessments. Furthermore, detailed hormonal profiling was not performed, despite the known sex-dependent regulation of adipokines; however, sex adjustment was applied in all analyses to account for this effect partially. Furthermore, potential selection bias should be considered, as participants were recruited from individuals seeking care at a single university-based periodontology clinic, and participation was voluntary. Therefore, this may limit the external validity of the findings. Additionally, oral hygiene habits were similar among the study groups. This suggests that variations in oral hygiene were unlikely to substantially influence the observed associations. Information on alcohol consumption was not systematically recorded and should be considered as a potential limitation when interpreting the findings. Accordingly, the present findings should be interpreted as associative rather than causal. Future age-matched longitudinal studies incorporating comprehensive metabolic, hormonal, and lifestyle assessments are warranted to elucidate further the role of asprosin in the interplay between obesity and periodontal inflammation.

Within the limitations of this cross-sectional study, the present findings support the study hypothesis by demonstrating that asprosin levels vary according to obesity and periodontal status. Higher GCF and serum asprosin levels in obese individuals, as well as increased serum asprosin levels in normal-weight individuals with periodontitis, suggest that both obesity and periodontal inflammation may be associated with circulating and local asprosin levels. In contrast, TNF-α and IL-6 showed less consistent patterns, indicating that traditional inflammatory biomarkers may not uniformly reflect the combined effects of obesity and periodontal inflammation. The observed associations between asprosin, obesity, and periodontal inflammation should be interpreted cautiously due to the observational design and potential sources of bias, including selection bias. Further longitudinal and mechanistic studies are required to clarify the role of asprosin in the relationship between obesity and periodontal inflammation. 

## Supplementary Information

Supplementary Material 1.

## Data Availability

The datasets used and/or analysed during the current study are available from the corresponding author on reasonable request.

## Abbreviations

ADA: American Diabetes Association
BOP: Bleeding on probing
BMI: Body mass index
CAL: Clinical attachment level
EFP/AAP: European Federation of Periodontology / American Academy of Periodontology
ELISA: Enzyme-linked immunosorbent assay
FBG: Fasting blood glucose
GCF: Gingival crevicular fluid
GI: Gingival index
G*Power: G*Power statistical power analysis software
HbA1c: Hemoglobin A1c
IBM SPSS: IBM statistical package for the social sciences
IL: Interleukin
IL-1β: Interleukin-1 beta
IL-6: Interleukin-6
IL-8: Interleukin-8
IL-10: Interleukin-10
IQR: Interquartile range
MCP-1: Monocyte chemoattractant protein-1
NH: Normal-weight periodontally healthy
NP: Normal-weight periodontitis
OD: Optical density
OH: Obese periodontally healthy
OP: Obese periodontitis
PBS: Phosphate-buffered saline
PI: Plaque index
PPD: Probing pocket depth
STEMI: ST-elevation myocardial infarction
T2DM: Type 2 diabetes mellitus
TNF-α: Tumor necrosis factor-alpha
VEGF: Vascular endothelial growth factor
WHO: World Health Organization
