# Supplementary material for: Serum and gingival crevicular fluid asprosin levels in obese and normal-weight individuals with and without periodontitis: a cross-sectional study
Source: BMC Oral Health. 2026 Apr 6;26:950. doi: 10.1186/s12903-026-08249-y (PMC13234963; doi:10.1186/s12903-026-08249-y)
Supplement: Supplementary file 1 — Supplementary Material 1. [file 12903_2026_8249_MOESM1_ESM.docx]

**Supplementary Table 1:** Median (IQR) Levels of Asprosin, TNF-α, and IL-6 in Gingival Crevicular Fluid (ng/30 s) and Serum (ng/ml) Across Study Groups (NH, OH, NP, OP)

| Variables (Median, IQR) | | NH (N=15) | NP (N=15) | OH (N=15) | OP (N=15) | **p** |
| --- | --- | --- | --- | --- | --- | --- |
| GCF Total Biomarker Levels (ng/30 s) | Asprosin | 1.03 (1.66)  [1.97- 2.76] | 2.63 (1.11) **^a^**  [2.21- 5.37] | 3.36 (1.39) **^a^**  [1.79-5.14] | 3.73 (1.58) **^a^**  [2.78- 6.91] | **<0.001^*^** |
|  | TNF-α | 0.09 (0.05)  [0.02-0.12] | 0.1 (0.01) **^a^**  [0.08-0.14] | 0.09 (0.03)  [0,07-0,14] | 0.1 (0.04) **^a^**  [0.07- 0.18] | **0.008^*^** |
|  | IL-6 | 0.03 (0.02)  [0.02-0.04] | 0.04 (0.01)  [0.03-0.05] | 0.04 (0.01)  [0.03-0.04] | 0.04 (0.03) **^a^**  [0.03-0.09] | **0.038^*^** |
| Serum Biomarker Levels (ng/ml) | Asprosin | 37.4 (13.11) [27.49-49.37] | 64.8 (31.27) ^a^  [35.80-108.90] | 82.4 (18.6) **^a, b^**  [70.48-103] | 73.85 (28.53) **^a,b^**  [42.74-104.5] | **<0.001 ^*^** |
|  | TNF-α | 0.22 (0.15) [0,09-0,55] | 0.19 (0.20)  [0,11- 0,36] | 0.31 (0.84)  [0,11- 0,89] | 0.35 (017)  [0.16- 0.42] | 0.081 |
|  | IL-6 | 0.16 (0.11)  [0.08-0.29] | 0.15 (0.09)  [0.10- 0.30] | 0.26 (0.27)  [0.11-0.53] | 0.22 (0.13)  [0.12- 0.34] | 0.05 |

Values are presented as median (interquartile range) unless otherwise stated. Group comparisons for continuous variables were performed using the Kruskal–Wallis test. When a significant overall difference was detected, pairwise comparisons were conducted using Dunn’s test with Bonferroni adjustment. Different superscript letters indicate statistically significant differences between groups (p < 0.05).

n: sample size, data are presented as median and interquartile range (IQR) and minimum–maximum [min-max].

*p<0.05 = statistically significant.

ᵃ = significantly different compared to healthy group; ᵇ = significantly different compared to normal weight periodontitis group; ᶜ = significantly different compared to obese healthy group.
